# A Study of Deployable Structures Based on Nature Inspired Curved-Crease Folding

**DOI:** 10.3390/polym16060766

**Published:** 2024-03-11

**Authors:** Gaurab Sundar Dutta, Dieter Meiners, Gerhard Ziegmann

**Affiliations:** Institute of Polymer Materials and Plastics Technology, Clausthal University of Technology, Agricolastr. 6, 38678 Clausthal, Germany; dieter.meiners@tu-clausthal.de (D.M.); ziegmann@puk.tu-clausthal.de (G.Z.)

**Keywords:** folding, curved crease, deployable structures, biomimicry, parametric modeling, visual programming, 3D printing, composite systems

## Abstract

Fascinating 3D shapes arise when a thin planar sheet is folded without stretching, tearing or cutting. The elegance amplifies when the fold/crease is changed from a straight line to a curve, due to the association of plastic deformation via folding and elastic deformation via bending. This results in the curved crease working as a hinge support providing deployability to the surface which is of significant interest in industrial engineering and architectural design. Consequently, finding a stable form of curved crease becomes pivotal in the development of deployable structures. This work proposes a novel way to evaluate such curves by taking inspiration from biomimicry. For this purpose, growth mechanism in plants was observed and an analogous model was developed to create a discrete curve of fold. A parametric model was developed for digital construction of the folded models. Test cases were formulated to compare the behavior of different folded models under various loading conditions. A simplified way to visualize the obtained results is proposed using visual programming tools. The models were further translated into physical prototypes with the aid of 3D printing, hybrid and cured-composite systems, where different mechanisms were adopted to achieve the folds. The prototypes were further tested under constrained boundary and compressive loading conditions, with results validating the analytical model.

## 1. Introduction

### 1.1. Early Motivation

Folding a very thin 2D sheet made of a paper-like material along a crease results in a 3D shape. In mathematical terms, this is called a developable surface where via certain operations a plane can be transformed into a smooth surface in three-dimensional space and vice versa without any distortion (e.g., stretching or tearing). There are various operations to yield a developable surface like bending, rolling, cutting, glueing and folding. This work focused on the study of folding behaviors.

While folding can be observed in many scales and shapes in nature, it was not very common in human civilization until the innovation of paper. The low thickness of paper and its ability to retain its shape once folded, made it very popular for such transformations compared to other thin flat material surfaces. Moreover, a small ratio of thickness over size of the paper enabled bending along folding lines without any stretching within the in-plane fabric [1].

Although there are assertions that folding existed in China and Japan as a cultural aspect since the introduction of and development in the quality of paper, concrete evidence of paper folds can be traced back only to the late 1600s [2]. Folding paper had a sacred meaning in ancient Japan and was called origami, concatenating *oru* (fold) and *kami* (paper). Since paper was extremely expensive at that time, origami models were mostly limited to religious and outstanding events. It was only during the twentieth century, with the improvements in paper production technology, that origami spread across the whole of Japanese society and eventually worldwide and became a tool of abstraction in art, design and architecture.

**Figure 1:**
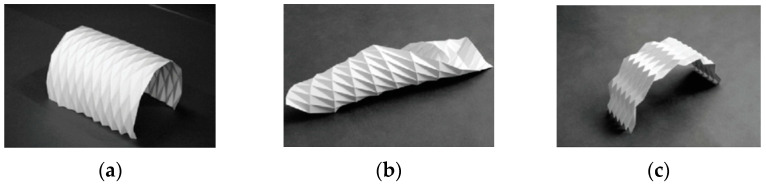
Three basic straight-crease Origami examples: (**a**) Yoshimura/diamond pattern, (**b**) diagonal pattern and (**c**) Miura Ori/Herringbone pattern [3].

Traditional paper folding mostly uses straight creases. In these models, the straight creases surround planar facets and comprise a polyhedral surface, hence they are also known as prismatic origami. Three basic patterns exist in this kind of folding, which is particularly popular in architecture and structural applications: the Yoshimura pattern, the Miura Ori pattern and the diagonal pattern [3] as can be seen in Figure 1. They use different combinations of folds and reverse folds to form simple curved surfaces.

**Figure 2:**
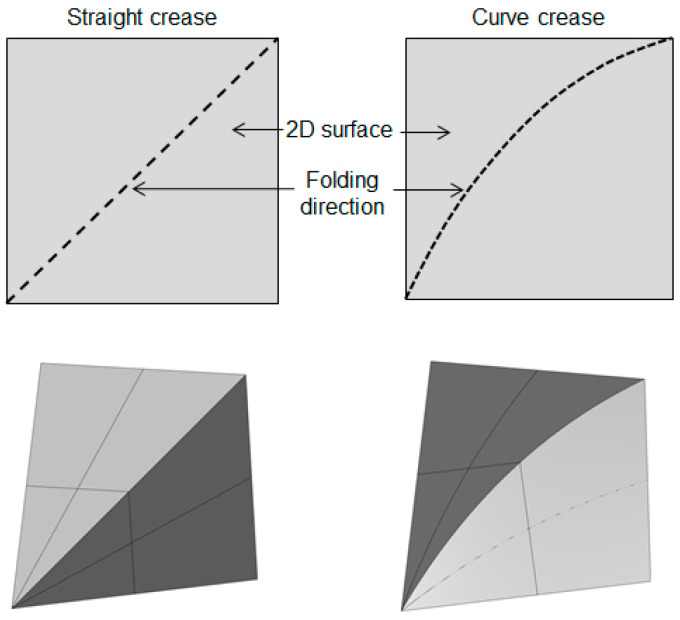
Comparison between straight-crease and curved-crease folding.

Whereas, altering the crease pattern into a curved fold, as illustrated in Figure 2, the same surface converts into a complex three-dimensional form with a hybrid of folding and bending with one side of the fold undergoing compression, while the other experiences tension along the surface.

Those hybrid characteristics make curved-fold origami not only a source of inspiration in architectural design, but also a significant tool in structural science and form-finding, specifically in shell structures. In general, shells are spatially curved surfaces supporting external applied loads. They normally consist of very thin (high ratio of width to thickness) sheets and hence are flexible towards bending and are used for efficient façade and roof designs. To cover a given area by a roof, engineers would conventionally use column-based models, which are beams repeated at some systematic spacing for structural stability mainly taking bending and shear into consideration. Consequently, the number and sizes of the beams increase as the area to be covered is increased, thus making the set-up aesthetically unpleasing and economically expensive. Alternatively, to cover the same area, a thin shell generated from a flat surface would take tension and compression into account and would result in a more economical and aesthetic solution. The mechanical advantage of shells over beams makes them a preferable choice for extremely lightweight and wide-spanned constructions with large weight-carrying capability. While conventional shells are evolved by bending flat surfaces, using curved creases as folding patterns, the same surface can evolve different forms of shells and gives much more flexibility for free-form deployable structure generation. This work tried to investigate test cases related to curved folding to establish a methodology for finding the right curve.

### 1.2. Brief History of Curved Folding

**Figure 3:**
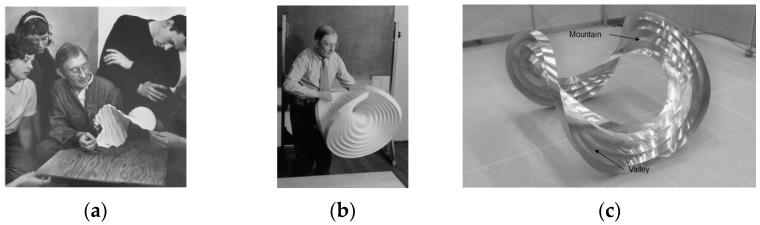
(**a**) Josef Albert with his students at paper folding course [5], (**b**) Josef Albert with one of the early curved-crease folded models comprising back-and-forth concentric circular folds [6], (**c**) a Bauhaus design model with definitions of mountains and valleys [7] (Pictures reproduced with permission).

The first instances of curved folded structures were developed by the students of Bauhaus in the 1920s under the supervision of Josef Albert, where the models were essentially spatial shapes developed out of thin paper via concentric circular folds with alternating mountains and valleys [4]. The initial idea was only to develop different shapes, rather than focusing on the functional requirements, with materials being the only constraint. Some construction examples can be seen in Figure 3. This methodology led to the understanding of structures based on the aspect of design. Thus, a material could be used more efficiently, leading to lightweight constructions.

**Figure 4:**
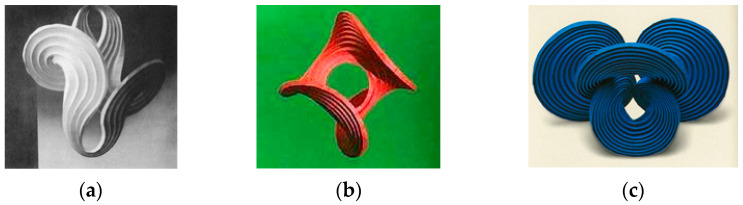
(**a**) Irene Schawinsky’s concentric circle model, (**b**) Thoki Yenn’s “Before the Big Bang” model, (**c**) Kunihiko Kasahara’s model of “Extreme Origami” [8] (Pictures reproduced with permission).

The distinct feature of these models was a hole that the forms were built around. Later years saw derivation of this idea explored by scholars like Irene Schawinsky, Thoki Yenn, Kunihiko Kasahara, etc., who modified the shapes into more complex 3D forms with larger holes and varying geometries [8], as shown in Figure 4.

**Figure 5:**
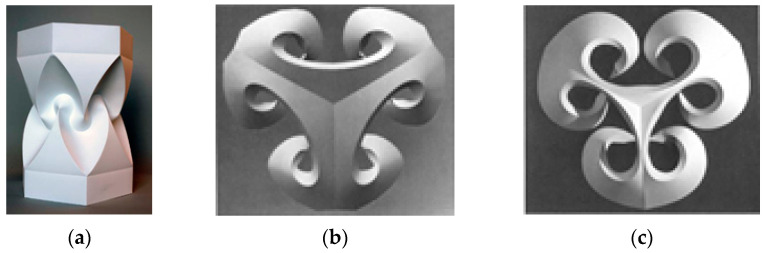
(**a**) One of the celebrated works of David Huffman “Column with Cusps” reconstructed by Duks Koschitz [10], (**b**) computer-simulated image, (**c**) physical model of Ron Resch’s developable surface model named “The White Space Curve Fold with 3-fold Symmetry” [11] (Pictures reproduced with permission).

Beyond the Bauhaus model and its successors, the most significant works in curved-crease folding before the computational era probably came via the works of computer scientist David Huffman and artist Ron Resch. Both contemporary innovators, although they worked on the same theme and often had many discussions, they approached the problem differently. While Resch was interested more in applied techniques for curved-crease folded sculptures and went on to realize some of his works as computational models, Huffman followed the analytical route to develop a mathematical understanding of folding [9,10]. Figure 5 illustrates some of their popular folding models.

**Figure 6:**
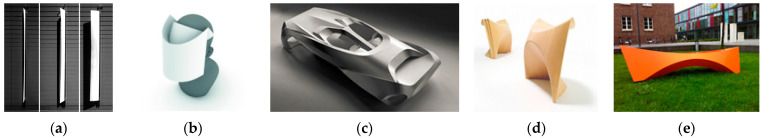
A few examples of curved-crease folded forms as use cases: (**a**) Prototype of a façade shading lamella based on ‘Flectofin’ design inspired from the deformation in the Strelitzia reginae flower [21], (**b**) a curved-crease origami face shield computer model [25], (**c**) ‘Bentley Tailor Made’ project by designer Kyungeun Ko in collaboration with RoboFold [26], (**d**) ‘Sit’—a stackable stool made of plywood designed by Andreas Lund [27], (**e**) the ‘Colonna Curva’ installation by Marco Hemmerling and Alessio Mazzucchi [28] (Pictures reproduced with permission).

Now, with developments in computer-aided design and manufacturing, curved-crease folds are no longer limited to paper sculpture art but have expanded into design and architectural examples with materials like thin metal sheets being used as shell structures utilizing forms as structural components. Computational simulation tools like finite element modeling and visual programming also enabled designers to create folded shapes in the digital space and observe deformations before actual manufacturing [12]. However, due to the cumbersome nature of the process of simulation, preliminary design changes often involve a to-and-fro exchange of information between physical models and digital counterparts [13]. Advancements in automation have also paved ways to achieving such complex digital structures as physical prototypes from one single surface by using a robot arm to fold metal sheets around predefined crease definitions. Oscar Niemeyer’s ‘Cathedral of Brasilia’ [14], Gregory Epps’s works with RoboFold [15], Zaha Hadid’s ‘Arum’ [16], Tal Friedman’s origami pavilion [17], Patkau Architects’ ‘One Fold’ [18], etc., are a few notable examples in this field, as can be seen in Figure 6.

Mimicking nature also enabled designers to achieve the dynamic transformation of curved creases from a flat state and take advantage of intermediate states leading to the end shape [19,20,21]. The introduction of additive manufacturing in digital fabrication aided the expansion of similar ideas into the polymer domain, providing the design process with flexibility [22,23,24,25].

**Figure 7:**
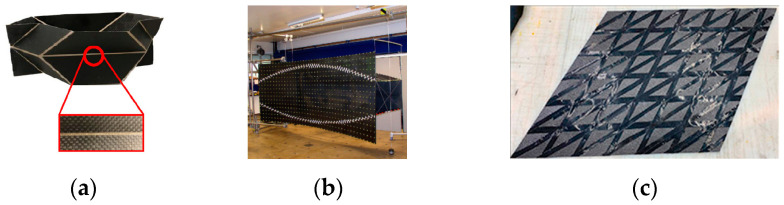
A few examples of folded deployable forms in fiber-reinforced composites: (**a**) Layup schematics of a foldable composite where the fold along the hinge is obtained by having two rigid traditionally cured carbon fiber-reinforced facets squeezing a dry layer of fiberglass impregnated with a very flexible epoxy resin system [29], (**b**) a woven fiber deployable antenna reflector demonstrator in deployed configurations [30], (**c**) an architecturally stiffened composite made up of dry fiber fabric and prepreg [31] (Pictures reproduced with permission).

In recent times, there has also been a surge of interest in the field of fiber-reinforced composite systems toward achieving deployability through various mechanisms. While linear-crease folds are the predominant approach due to the complexities associated with realizing curved structures using anisotropic materials, there are noteworthy instances within the literature, as illustrated in Figure 7, that explore the potential of curved-crease folds in applications involving fiber composites.

### 1.3. Digital Tools for Curved-Crease Folding

As mentioned in the previous section, although folded structures have been investigated extensively in art, architecture and engineering, the methodology to virtually design a shape with curved folds remains a difficult problem due to limitations in tools for digital construction. Most developments in the digital frontier in recent years proceeded in two ways: replicating equivalent forms of paper models in the digital space and investigating the effects of folding in the structure.

**Figure 8:**
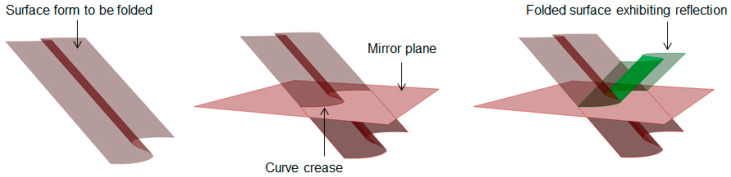
Mirror reflection pattern evolving curved folding.

Simplifying the problem by limiting the curve to be planar and investigating the performance of the evolved form against a subjected test case reduces the complication drastically for the designer. When the curve under consideration is contained in a single plane, referring to the research of David Huffman, a pair of generating lines at any point on the fold make equal angles with that plane. This means that the plane acts as a mirror plane that reflects the tangent plane on one side to the tangent plane on the other side [32,33]. Hence, it can be safely assumed that a shape generated by applying mirror reflection to part of an origami surface is also an origami surface. A simple construction of such mirror reflection can be seen in Figure 8. By assuming that different reflective mirror planes do not intersect each other, a complicated origami surface with planar curved folds can be obtained by applying multiple reflections to a simple origami surface.

Further development in the analytical computation of curved shapes took place in recent years. Fuchs and Tabachnikov [34] described a few fundamental observations from their experiments to describe the stability of a curved fold and consequently justified the arguments by applying mathematical theorems. Kergosien et al. [35] proposed a mathematical model for simulating the bending and creasing behavior of paper-like sheets, while Frey [36] developed a model to predict buckling. Kilian et al. [37] developed a simple interactive model to describe multiple folding by adding new folds on a folded surface by virtue of discretization, while Mitani and Igarashi [33] described a tool to add a sequence of folds to a digital surface interactively.

Among commercial software, Freeform Origami© ver 0.1.5 Alphawas one of the first simulation programs to design deployable structures based on curved-crease folding developed by Tomohiro Tachi [38,39]. It used discretization of crease patterns and provided interactive animation of the folding process. Kingkong [40], a plug-in for the visual programming platform Rhinoceros/Grasshopper has also been developed by RoboFold, which allows simulation and parametric control of curved folded models. It allows the smooth integration of a robotic manufacturing process into the design domain. Daniel Piker developed a component for the Kangaroo Physics [41] plug-in for Grasshopper called Origami which allows the user to define a mesh and assign the mountain and valley folds. More recently Crane [42], a Grasshopper plugin for the design and simulation of rigid folding origami, was developed by Kataro Tanimichi et al. Finite element tools like Karamba [43] and Sofistik [44] also allow the structural simulation of such forms under prescribed loading. Consequently, different simulation tools, depending on programming knowledge and problem requirements, provide different possibilities to designers [4].

## 2. Materials and Methods

### 2.1. Basic Design Setup

**Figure 9:**
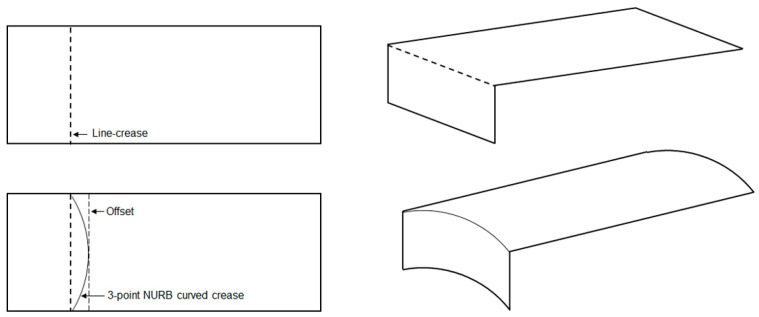
Illustration of initial case-study setup with offset definition.

Since the objective of this work was to find an optimal curve of fold for a deployable shape from a structural deformation perspective, a design problem had to be formulated first to approach the issue. For this purpose, an overhanging roof, as illustrated in Figure 9, was considered as the starting point which can be developed via a folding operation from a simple rectangular shape made out of a very thin paper-like material. In other words, the idea was to apply folding to the planar surface at a certain distance so that, when folded and held in an upright position, the evolved 3D shape can work as an extended roof. Moreover, when one edge is kept fixed to the ground, this shape should also be able to withstand self-weight as well as external loading conditions while maximizing the shade area underneath. For a line crease, the execution of such a setup is relatively straightforward where a line segment passing through the surface can be defined as the crease pattern and a 3D shape can be generated. Assuming this as the base model, the end points of the line crease can be taken as inputs for the generation of desired curved crease. Another line parallel to the existing line crease can be assumed with an *offset* distance, and the mid-point of the newly created line segment, along with earlier points, can provide the most basic definition of a NURB curve, resulting in a curved-crease model.

### 2.2. Parametric Model for Folding

**Figure 10:**
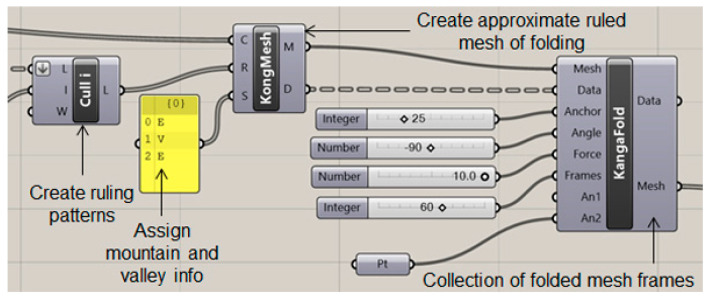
Parametric modeling of folding in Grasshopper with Kingkong toolboxes.

To generate a digital model of the setup explained in the previous section, parametric modeling in Grasshopper was used with the Kingkong plug-in and the Karamba shell element finite element toolbox. The basic working principle of the Kingkong plug-in is described in Figure 10.

**Figure 11:**
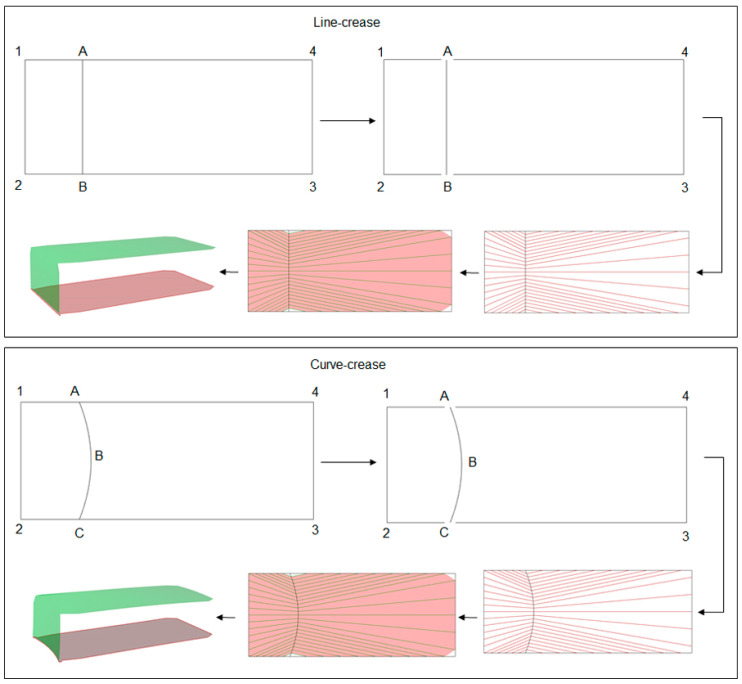
Step-by-step development of folded models based on a line and curved crease.

The initial curve definitions constituting the geometries as described in Figure 9, serve as the input for the plug-in. For example, in the case of line-crease folding, the assumption is that the line *AB* folds the surface *1234* into a 3D shape as shown in Figure 11. Hence, the input open-curve combination would be *A12B*, *AB* and *A43B*, while, for a curved-crease fold *ABC*, the same combination of curves would be *A12C*, *ABC* and *A43C*. Based on the inputs, the plug-in automatically creates arbitrary evenly distributed ruling patterns. These rulings work as the guide to generate a mesh using KongMesh toolbox. Here, the user is required to provide the mountain and valley definitions to the intended fold. The generated mesh essentially approximates the original geometry and hence some information along the fold edge is lost in the process. This can further be reduced by making a finer mesh with more elements, which in turn increases the computation time.

The generated mesh is then subjected to the KangaFold toolbox, which, based on other parametric inputs, produces a collection of frames from the start to the end position of the folded shape. For the present test case a 90-degree fold was chosen. The folded shape was then adjusted according to the problem definition and a shadow of the shape was generated on the ground plane, which would essentially demonstrate the functionality of the shape as a roof structure.

**Figure 12:**
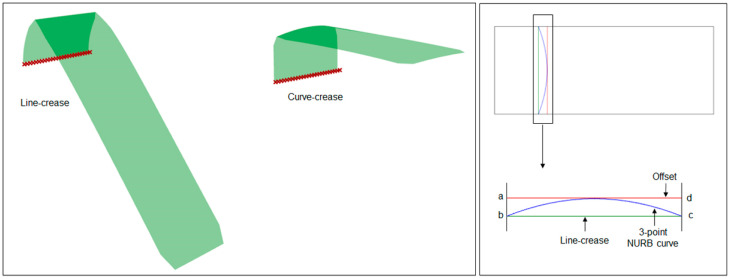
Equally scaled deformation state of the models simulated using the Grasshopper Karamba plugin for self-weight with one edge being fixed and the representative design space definition *abcd* with a 3-point NURB curve as one potential curve of folding.

From the design point of view, it is evident that the line-crease folded shape would have a maximum area covered underneath, as in the case of curved-crease folding some area information is superimposed to accommodate bending along the surface. But, due to the spring-like bending behavior along the curved surface, it can be assumed that curve-crease model would withstand loading better as compared to its line-crease counterparts. A simplified finite element analysis using the Karamba plug-in for deformation due to self-weight validates this assumption as illustrated in Figure 12. Evidently, the offset between the line and curve crease would play an important role in optimizing a final shape.

It is also important to note that these assumptions were true for all the material inputs available inside the Karamba material library, as long as the surfaces in consideration were designed as homogenous geometries. For ease of computation, material properties for all consequent simulation models were kept constant at the isotropic ‘wood’ input [45].

Thus, based on the design criteria, the functionality of the deployed shape, and the dimension of the shapes, an optimization problem can be formulated within the domain of parametric modeling, which takes care of each objective and produces a solution for a given offset distance. Consequently, a design space can be created with the line-crease definition and the offset limit being the constraint values, such that any evolved curve should lie inside this region, thus reducing the geometry optimization problem into an intermediate free-form curve evaluation process.

### 2.3. Looking for Inspiration in Nature

Biological forms have been a major motivation in human intellectual development since the early ages. Natural forms are optimized for a given environment. Mimicking these forms and their functionalities yields solutions which often remain unnoticed in conventional methods. This process of mimicking or copying biological principles is often referred to as ‘biomimicry’ which is an established area of investigation in contemporary architecture and engineering [46,47]. Depending on the scale and application, there exist different levels of biomimicry which often involve inspiration from a particular set of biological forms. For this work, inspiration was derived from the growth of plants in branching direction [48] and the idea was to mimic the form to evolve a curve for folding.

**Figure 13:**
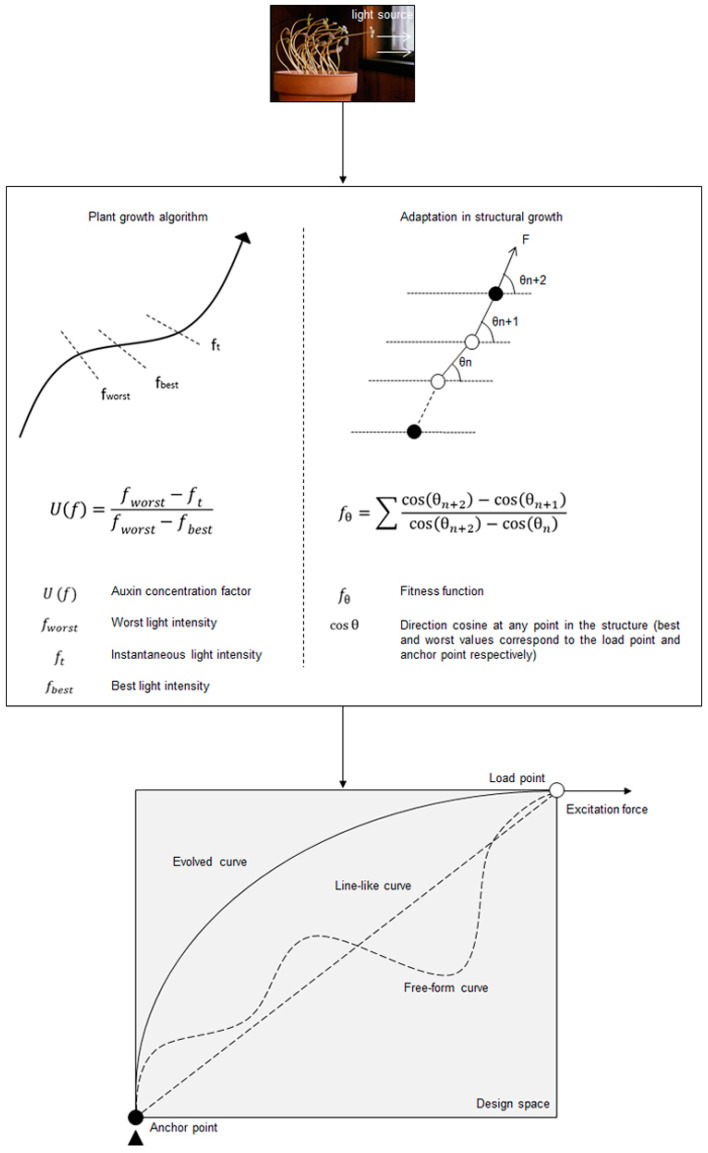
Schematic representation of a 2D form-finding process taking inspiration from nature by correlating a plant-growth algorithm with a representative growth intensity factor.

In plants, the growth mechanism is understood to be driven by the concentration of auxins, a hormone regulating cellular growth and coordinating the emergence of plant geometry. The auxins are triggered mostly by the availability of resources and, in the case of branching, it is sunlight that saturates exposed auxins to enable bending [49,50]. Here, the analogy between auxin and light was translated into a form–force relationship and the evolved forms were then optimized via an evolutionary programming toolbox in Grasshopper. A brief explanation of the step-by-step development of the curve evaluation process is illustrated in Figure 13. Depending on a given excitation force input and control point definitions, the solver would generate random free-form curves and would try to minimize the fitness function. In previous works [51,52], the idea was successfully implemented to evaluate the optimal form of a curve between two anchor points, oriented along the direction of an excitation force applied at one anchor point, with the other one being fixed. Thus, complex 3D free-form shapes were generated resulting in efficient load-bearing structures.

**Figure 14:**
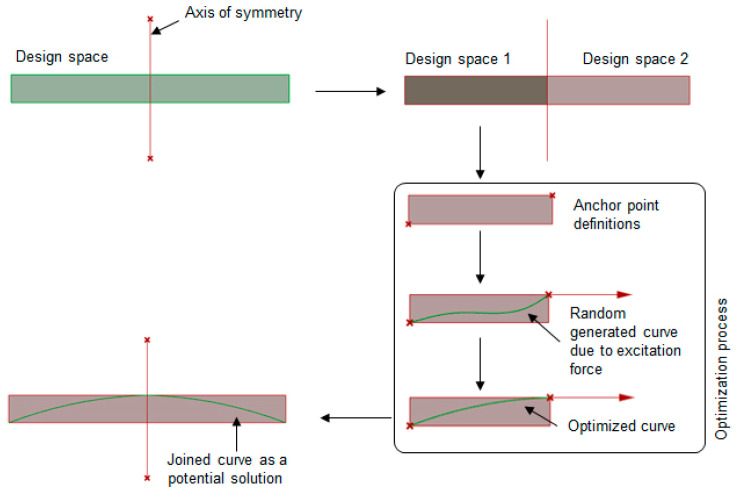
Step-by-step explanation of the process of evolving a curve by taking inspiration from a plant growth mechanism.

Extension of the stated optimization process into the present work required a few more modifications. Firstly, the mentioned analogy was constrained to a 2D design space. Then, two separate design spaces were created from the original space mentioned in Figure 12 via an axis of symmetry as shown in Figure 14. Two diagonally opposite points in each design space represented the anchor points within which a curve of evolution would be generated. Once evolved, both curves from two design spaces were joined to create the desired curve for folding.

**Figure 15:**
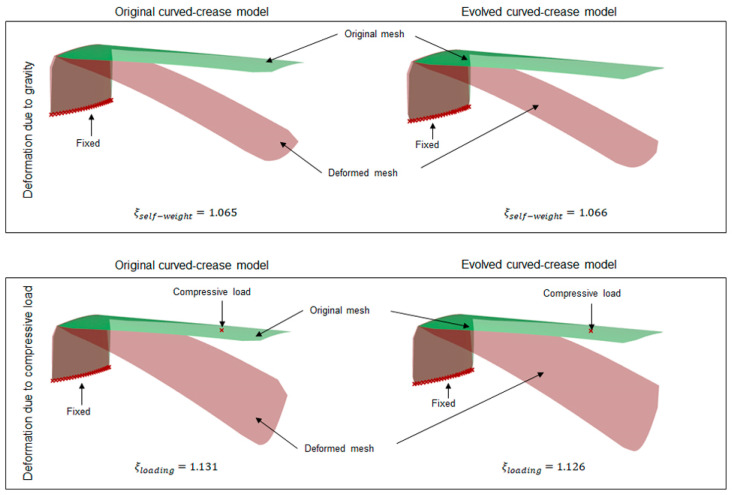
Equally scaled mesh deformation comparison between the original 3-pt NURB curved-crease model and the evolved curved-crease model with the corresponding deformation coefficient *ξ* for self-weight and user-regulated compressive load at a random point on the overhanging face of the shape.

Once the methodology for evaluation of curves was established, the next step was to generate folded geometries from the respective curves and compare them with standard three-point NURB curve solutions. For this purpose, as a representative model, a rectangular surface with a dimension of 5 cm × 12 cm was chosen with line-crease at a distance of 2.5 cm from the edge, with a minor offset distance of 1 cm, thus making a design space with dimensions of 5 cm × 1 cm. Both the NURB curve and the evolved curved crease models were tested for self-weight and random compression load scenarios. A parametric model allows the user to easily interact with the geometry in run-time for multiple inputs. Consequently, a multipoint or multidirectional force input for such a case can easily be formulated. For simplicity, only the self-weight and compression force at one single point on the bent surface are displayed here. Thus, a more complex load scenario can be assumed to be an extension of such a case. To streamline the visualization of the results in Grasshopper, a factor *ξ*, as a deformation coefficient, was introduced [52]. This coefficient essentially represents the ratio between the deformed mesh area after loading and the original mesh area. By using the coefficient, initial insights into shape deformations could easily be estimated without requiring specialized technical knowledge to interpret stress values. Figure 15 provides a summary of the simulated results obtained from the parametric mode.

It can be inferred from the results, that the evolved curved-crease model exhibits slightly superior performance when contrasted against the original three-point NURB curve model. The minimal disparity in deformation coefficient values can be attributed to the minor offset, resulting in a smaller design space. As a consequence, both curves nearly align with one another. However, it is worth noting that this alignment may not persist in scenarios involving multiple folds or more substantial offsets. To delve deeper into this concept, new test cases were devised featuring instances of multiple folds.

### 2.4. Multiple Folds and Prototyping

Integrating another valley fold mirroring the original fold at the other end of the previous model transforms the model into a shape supported at two ends instead of an overhanging structure. The rationale behind developing such folded shapes was the ease of physical prototype testing where two ends of such geometries could be kept fixed and a compression load could be applied to the bent surface.

**Figure 16:**
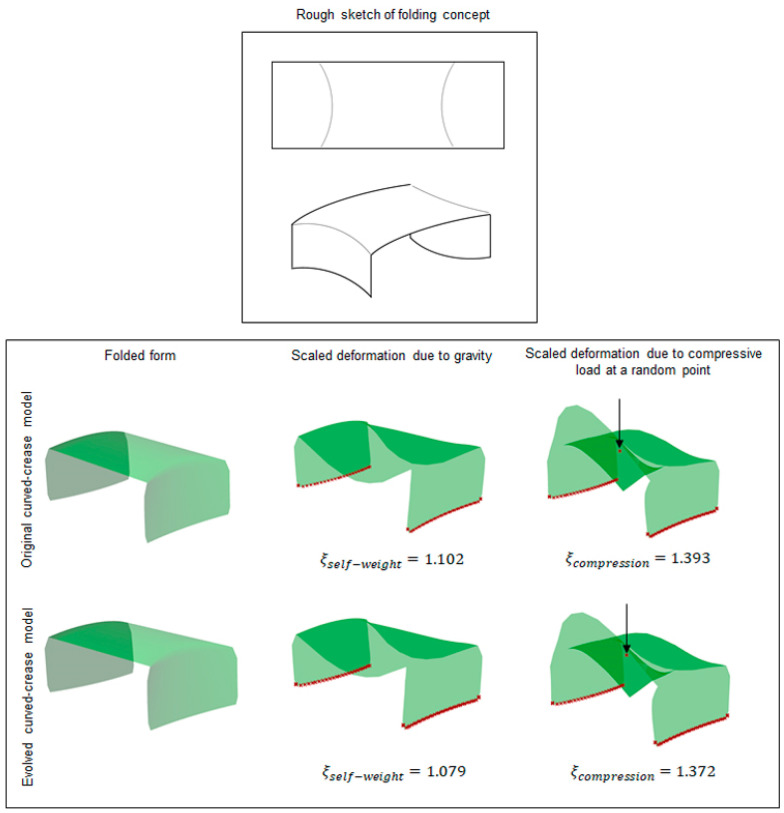
Rough sketch of two valley fold test case setups with deformation results obtained via Grasshopper analysis.

Similar to the previous test case, the generated models were tested under their self-load and the user-defined compressive load at a random point. As can be seen from Figure 16, introducing multiple folds already reflects a significant difference between the simulation results in terms of the deformation coefficient value.

**Figure 17:**
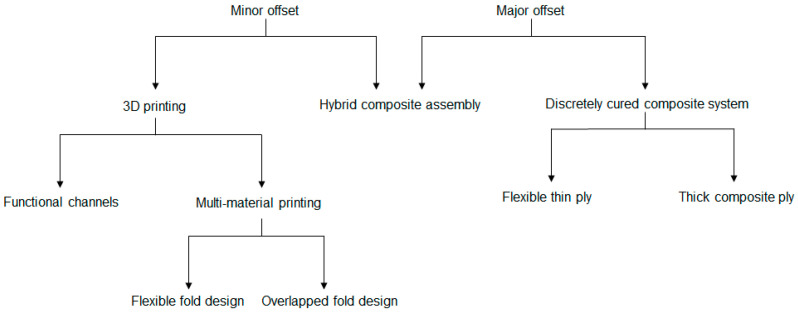
Different methods adopted for prototype construction and validation.

To validate the hypothesis developed within the parametric model, different prototypes were conceptualized, each with different mechanisms to realize the folds. The objective was to have a qualitative understanding of the scope of the developed model and its limitations based on output results. Thus, prototypes were constructed for different scales and physical and material properties to test the robustness of the parametric model, as well as to explore the transportability of the concept between various manufacturing processes. Moreover, to cover a broader spectrum of folding, minor and major offset case studies were taken into consideration. Figure 17 briefly summarizes all the experimental designs considered in this work, which is followed by discussion of individual manufacturing processes.

#### 2.4.1. Three-Dimensional Printed Prototypes

Three-dimensional printing was chosen as the first manufacturing method because of the ease of prototyping. Moreover, depending on the printer resolution, it is possible to produce accurate samples as described in the 3D models which was essential for minor offset prototypes.

**Figure 18:**
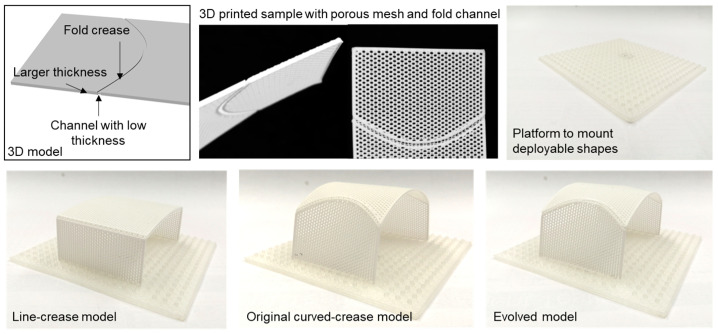
Design process and 3D printed models using the functional crease channel.

To realize the folds with functional channels using a single printing material, the geometries were modified by assigning a thicker surface (1 mm) with a thinner folding channel crease (0.5 mm). This provided discreteness and flexibility to the surfaces, which was necessary to perform folding without cracks. Moreover, the surfaces were designed as porous meshes to enable bending. An FDM printer (ANYCUBIC i3 Mega S, Shenzhen, China) equipped with an ABS [53] material was used to print the fold samples with an overall surface dimension of 5 cm × 12 cm. To test the samples, a platform with small probes was designed where folded shapes were held upright for compression loading. Details of the design process are shown in Figure 18.

**Figure 19:**
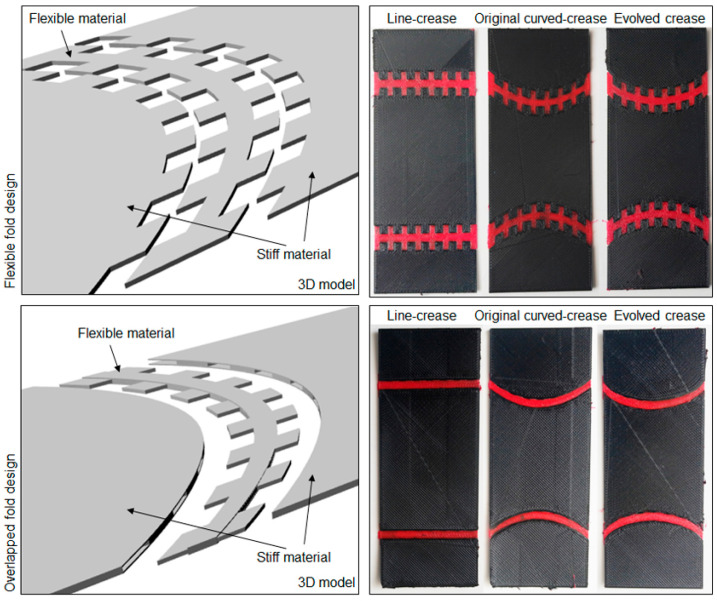
Slot mechanism designs for multi-material samples and printed prototypes.

The idea was further extrapolated to a multi-material printing process, where a flexible fold region was realized by a TPU [54] material with PETG [55] being the base material for the rest of the surface. Keeping the overall surface dimensions unchanged from last time, two types of folding mechanisms were investigated, one with repetitive slots throughout the thickness of the samples and another with slots to reinforce flexible regions with a stiff material at the top and bottom. Illustrations of fold mechanisms along with printed samples are described in Figure 19.

#### 2.4.2. Hybrid Composite Assembly

**Figure 20:**
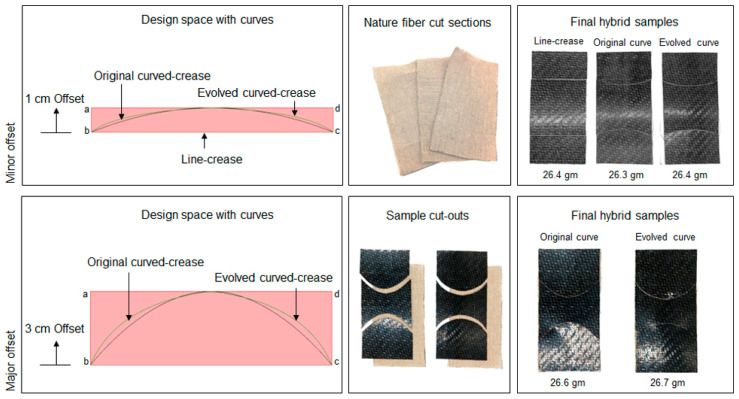
Design space definition *abcd*, material preparation and final hybrid composite samples for each crease definition for both minor and major offsets.

To extend the application of the setup into the composite domain, scaled-up models were chosen to be produced first using hybrid assembly. The idea was to establish a validation process for the 3D printed sample results which could be extrapolated, first to the major offset test case with a similar hybrid model that then could be tested with a discretely cured composite system. For this process, thin natural fiber textiles [56] of 0.5 mm thickness were cut into specific shapes of 10 cm × 20 cm and fold creases were marked on each of the surfaces, creating three sections on each surface. Then, pieces of exact dimensions from pre-consolidated glass fiber-polypropylene composite sheets [57] of 0.5 mm thickness were cut and glued onto each section separately. Common bioplastic-based glue [58] was used for this process and the samples were pressed for 24 h to achieve uniform adhesion between the layers. As mentioned earlier, the process was repeated for both a minor offset of 1 cm and a major offset of 3 cm. Thus, the obtained models, as shown in Figure 20, can be considered to be origami-inspired hybrid composites instead of true-origami models as the fold creases were realized by cutting instead of folding. Lastly, a special holder was also designed using thick PVC sheets [59] to provide the necessary boundary constraints for the samples.

#### 2.4.3. Discretely Cured Composite System

To achieve a balance between flexibility and stiffness within a single fiber-reinforced composite, a careful selection of resin systems was necessary. This involved utilizing a hard resin system for certain sections; while opting for a soft resin system for the folding regions. A combination of thermoplastic and thermoset resin systems was needed to achieve the goal.

**Figure 21:**
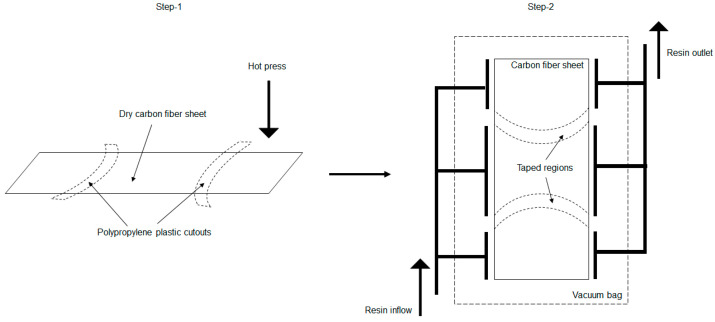
Illustration of two-stage impregnation process adopted for discrete curing of fibers.

Consequently, a two-step process was implemented where, in the initial stage, the folding regions were enveloped with Polypropylene (PP) sheets [60]. This arrangement, as illustrated in Figure 21, was then placed inside a hot press at a temperature of 160 degrees Celsius, thus allowing the PP to melt and potentially infuse through the dry fiber layers.

Subsequently, the setup was transferred to a vacuum assisted resin infusion (VARI) [61] configuration. The areas impregnated with PP were carefully shielded using insulated tape. The entire system was then vacuum-sealed into discrete sections to facilitate infusion of the thermoset resin [62]. After the infusion process was completed, the samples were positioned in an oven and left overnight at a temperature of 60 degrees Celsius to undergo curing before testing.

**Figure 22:**
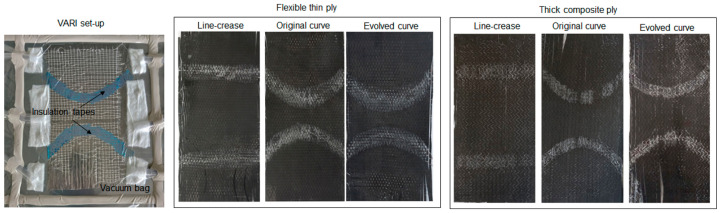
Discrete resin infusion setup for a sample and the final cured samples with the fold region infused with thermoplastic resin.

Material selection for the setup involved opting for unidirectional (UD) carbon fibers. The dimensions were matched with the scaled-up dimensions of the hybrid assembly. For the development of thin and thick ply prototypes for each fold crease, two distinct types of UD carbon fiber fabrics with plain weave areal weights of 270 g/m^2^ [63] and 320 g/m^2^ [64] were chosen, with thin ply fabric sheets woven transversely with the glass fibers, while the thick ply system was woven with standard textiles. To avoid errors due to manufacturing limitations, the test samples only for the major offset with a distinct difference between the curve creases were prepared. The actual set-up and one set of finished prototypes can be seen in Figure 22.

## 3. Results and Discussions

As mentioned earlier, once manufactured, all of the samples were subjected to compressive load tests at their respective final folded shapes. Two edges being fixed, the elevated surface was pressed with a specially designed probe. For each type of prototype, three samples were prepared for testing for repeatability and the results were aggregated to avoid errors. The loads were applied until either the elevated surface touched the platform due to bending or until breaking point. Forces in N induced in the system due to downward movement of the compression probe were plotted against the percentage of deformation in the systems. The obtained results were superimposed for all three models, namely: line, three-point NURB curve and evolved curve for each case separately as mentioned in Figure 16 in order to understand trends in the deformation for each folding mechanism.

### 3.1. Minor Offset Test Case

**Figure 23:**
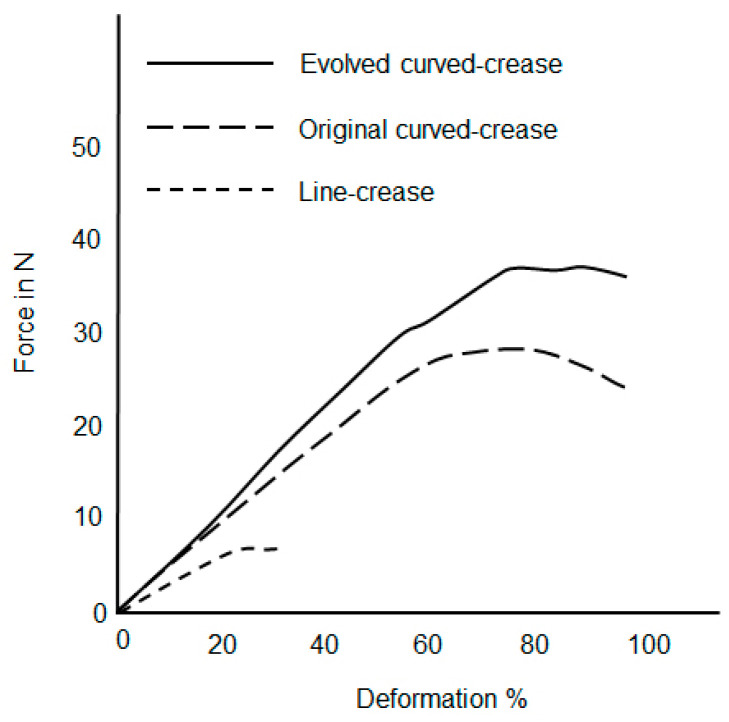
Aggregated force-deformation distribution for samples produced by 3D printing using an ABS material comprising functional crease channels.

Figure 23 depicts deformation results for samples with minor offset and functional folding channels prepared via 3D printing and as described in Figure 18. The nature of the deformation among the samples were as expected from the parametric model simulation, with the evolved curved-crease model exhibiting higher resistance to the applied load as compared to the original curved-crease model for the same amount of deformation, with the line-crease model being the weakest.

**Figure 24:**
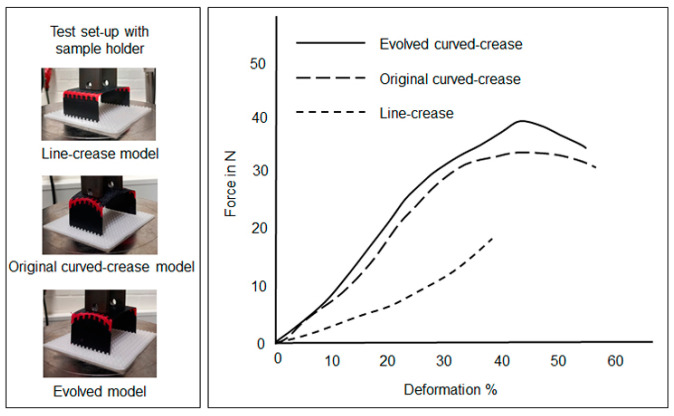
Deformation under loading in samples produced with PETG and TPU consisting of the flexible- and exposed-fold designs.

**Figure 25:**
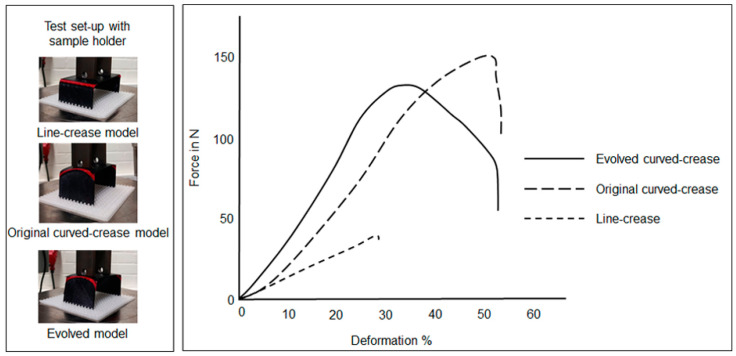
Deformation under loading in samples produced with PETG and TPU consisting of the overlapped-fold design.

In the minor offset test case discussion, the next set of prototypes were models printed with a tough and flexible material with different folding mechanisms as described in Figure 19. Figure 24 and Figure 25 reflect the results of the flexible and overlapped fold samples, respectively. It can be inferred from the figures that for the flexible and exposed fold, the trends in deformation are similar to previous results.

With the overlapped-fold design, the resistive response of all structures increases drastically for the initial phase of deformation due to the stiffer surfaces and joint mechanisms. But beyond a certain deformation range, the resistive force in the evolved curved-crease samples decreases sharply as compared to the NURB curved-crease samples. This is also due to the fold mechanism design, which at this point restricts the structure from stretching anymore along the folding direction. Hence, the resultant curved shape after a certain amount of loading transforms into a shape similar to the line-crease model. Since the form of the evolved crease is marginally flatter as compared to the standard three-point NURB curve, the transformation occurs earlier in such models. Additional investigation is necessary to enhance this type of fold mechanism setup by introducing flexibility through adjustments to various geometric parameters within the model.

**Figure 26:**
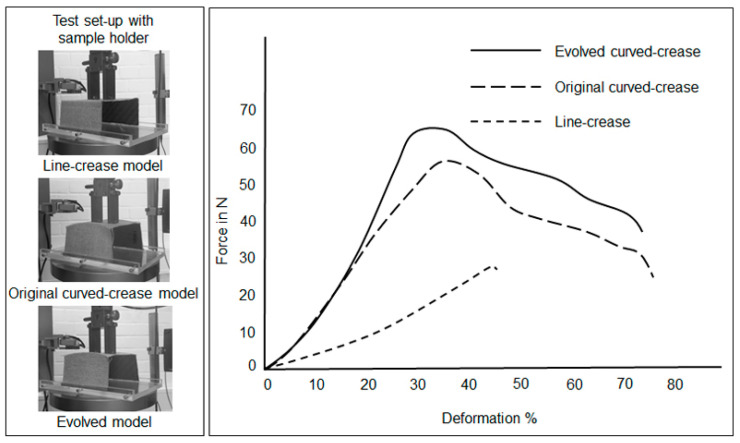
Compression loading results for samples produced by cutting and joining pre-consolidated fiber sheets.

The last set of test samples in the minor offset test case were hybrid composite assemblies. Figure 26 describes the force–deformation relationship for the samples under compression loading. The trends obtained in this test also validate the hypothesis that the evolved curved-crease form works marginally better as a load carrier in contrast to the other samples. It is noteworthy that, although these samples were scaled-up versions of previous models, the strengths in the system are lower compared to the multi-material 3D printed samples with an overlapped-fold design. This is because upscaling results in longer lever lengths, consequently increasing bending moments in the system. Moreover, the fold realization process adopted in this method enables cutting of pre-consolidated fiber sheets, thus impacting the overall integrity of the systems under loading.

But, regardless of the manufacturing issues involved, due to the simplicity of preparation, this method provided an easy way of transferring knowledge between minor and major offset test cases, scaled-up models, as well as transition between additive manufacturing and composite systems.

### 3.2. Major Offset Test Case

**Figure 27:**
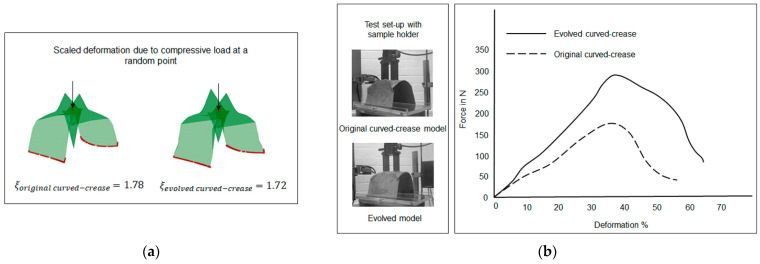
(**a**) Representative scaled-up deformation models obtained via Grasshopper analysis, (**b**) results obtained from compressive load testing of the major offset curved samples produced by hybrid assembly.

Before prototype testing for such test cases, the design space definition of the parametric model had to be readjusted. The model geometry definitions were changed according to the information mentioned in Figure 20. Similar to the previous test case, the models were designed in the visual programming domain and a random compression load input was introduced to the elevated surface in the final folded configuration of each model, with two edges being fixed. Figure 27a represents a scaled-up deformation model for the two curved-crease specimens under discussion along with the respective deformation coefficient values. The corresponding results of the compressive load test on the hybrid composite prototypes are described in Figure 27b. As can be seen, in both of the comparisons, the evolved crease model displays higher resistance against an applied load, i.e., less deformation against the same applied load value as compared to the original curved-crease model. Moreover, the obtained force values were higher in comparison with the minor offset prototypes as described in Figure 26. This is due to the fact that, with major offsets, the obtained curve forms are more acute, which dictates a higher amount of folding. Thus, resulting in larger bending among the specimens on the elevated surfaces.

**Figure 28:**
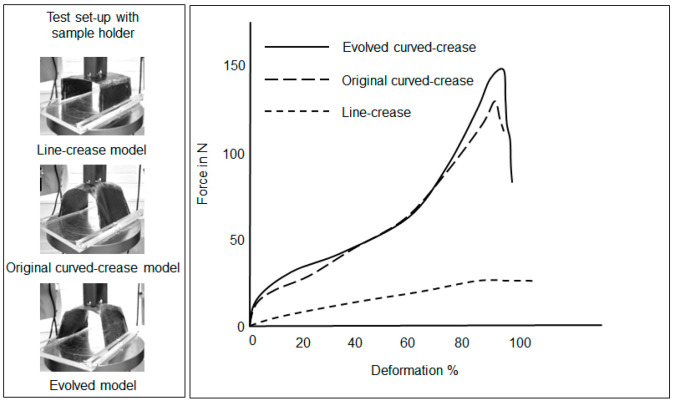
Force–deformation plot under compression loads for samples produced by discrete curing of thin ply unidirectional carbon fiber fabric.

**Figure 29:**
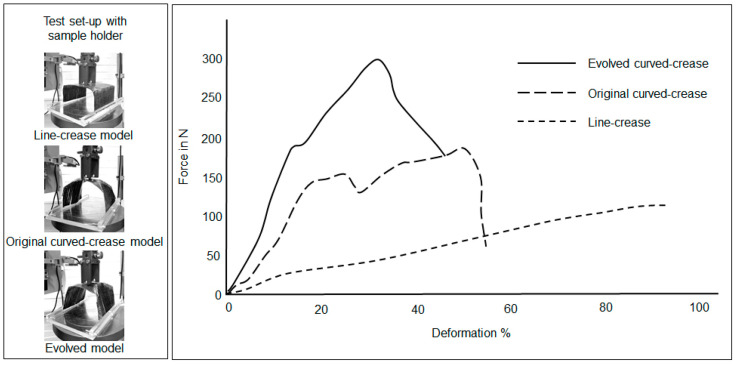
Force–deformation plot under compression loads for samples produced by discrete curing of thick ply unidirectional carbon fiber fabric.

The last section of prototyping involved discretely cured composites as described in Section 2.4.3. Figure 28 describes the results of compression tests for the thin ply samples while the thick ply sample results are plotted in Figure 29. For the thin ply system, it can be observed that the difference between the force–deformation plots for the original and evolved curved models is very minimal; with the latter form performing marginally better. This behavior can be attributed to the glass-fiber woven fabrics allowing stiffness in the transverse direction as well. The differences become drastic among the last set of samples with the thick ply, where deformation was dictated only by unidirectional carbon fibers. Furthermore, it is interesting to observe that the curved sample results of the thick ply composite system closely mirror those obtained for the hybrid assembly.

Once deformation trends observed within the developed parametric model were successfully replicated using different sets of prototypes, the model was also extended to address a few other curved-crease instances under similar boundary conditions as described in Figure 20, to understand the complete merit of the process. The corresponding deformation coefficient values are tabulated in Table 1.

It is interesting to note that, while the ellipse section has the largest curve span, the deformation is also largest because, in this case, the crease tends to create a linear fold instead of a curved one and thus the resulting structure does not exhibit elastic deformation along the surface to obtain stiffness. This is followed by the 3-pt circular arc where, again due to a larger perimeter, the folded shapes tend to deform more like a linear folding system. With the catenary curve, the model improves in terms of stiffness but for the same setup, the length of catenary curve is usually larger as compared to the 3-pt NURB or evolved curve. Hence, the folded model suffers comparatively higher deformation. Only the free-form evolved curve described in this work, despite having a higher curve length, produces a better geometrical configuration.

### 3.3. Short Discussion on Loading at the Folding Crease

**Figure 30:**
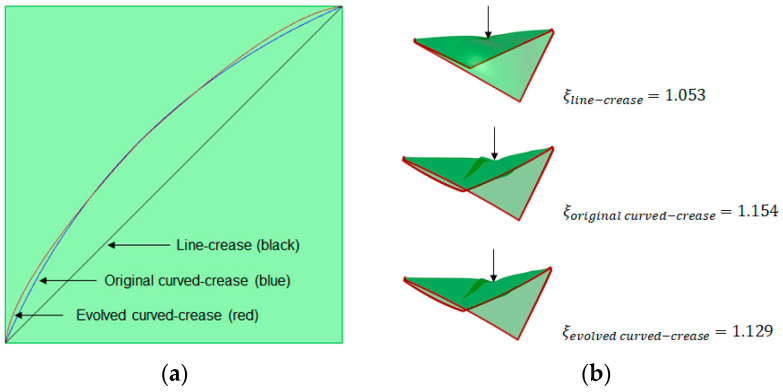
(**a**) Unfolded 2D surface with fold creases, (**b**) deformation due to loading at a point on the crease.

While crease folds provide an innovative way of form evolution, they also essentially weaken the structure exactly at the fold crease location due to plastic deformation. To realize this criterion in design domain, a test case was designed where the geometry of the piece of paper from Figure 2 was folded along the line curve, 3-point NURB curve and evolved curve with a minor offset and the folded shapes were simulated for loading at a random point strictly along the folding crease with other edges being fixed, as illustrated in Figure 30.

Interestingly, in this case, the line-crease model performs better under the compressive load as compared to the other two models. This is because, since the loading is exactly at the creased direction, the effect of elastic deformation along the folded surfaces could not play any significant role and the structures were reduced to a cantilever beam-like formation, constrained at two ends. Thus, the line crease acts like a straight beam and suffers less deformation compared to the curved ones. Even then, the deformation in the evolved curved-crease model was found to be lower compared to the original curved-crease model, due to minor but decisive differences in the radius of curvature between these two curved forms. This study provides sufficient evidence for taking additional care in designing and testing such models for loading along the folding direction.

## 4. Conclusions

While it is common nowadays to have folding mechanisms to be adapted into free-form structures for various purposes, curved folds remain a complex topic and require delicate attention. This is because curved-crease folds exhibit both plastic and elastic deformation to a deployable surface and modeling such forms encounters various logistical and design issues.

Thus, in digital modeling, simplification of the problem by limiting the curve to be planar and investigating the performance of the evolved form against a subjected test case reduces the complication drastically for the designer. Once limitations of the scope of folding and the digital tools are realized, the curved-crease folds can evolve fascinating and elegant structures which are significantly better in terms of performance, compared to their line crease counterparts.

A step-by-step development of the parametric model is described in this work using the Grasshopper visual programming toolbox. A novel method for generation of curves was discussed by taking inspiration from nature, following the growth mechanism in a plant and applying the analogy to finding curvature forms. Test cases were developed to evaluate the performance of the developed shapes in a digital space. The models were further modified to create distinct differentiation between the different curved-crease folds by modifying the offset distance. Throughout the design evaluation process, the material properties were kept as independent variables to enhance the transportability of the designed models to the physical prototyping domain across different manufacturing processes, scaling, and material usage. Consequently, the generated solutions were realized in the prototyping phase as 3D-printed samples, hybrid assemblies, and discretely cured composite systems with different folding mechanisms. The prototypes were subjected to compression tests, and the trends in the results obtained from the experiments were found to be mostly in good correlation with the simulation models. The overall performance of the evolved curved-crease models generated via biomimicry was found to be superior or marginally better compared to the other models under similar boundary and loading conditions.

However, the parametric model did not consider the stresses accumulated in the structure during the folding process. The digital tool also has computational constraints in terms of creating the approximate mesh by mapping the original surface and, hence, is limited to simple curved-crease models. Thus, in order to arrive at an accurate assessment of the effect of curvatures in the simulation domain, further investigation and improvements are required in terms of the parametric model. Furthermore, for a given manufacturing process and material definitions, the simulation results generated via the proposed model can also be validated using a commercial FE. Moreover, the different prototyping methods described here also have individual constraints, ranging from folding mechanism design to material and manufacturing limitations, and further improvements in specific fields will lead to better performing prototypes. While this study was limited to only two cases of offset prototypes, a detailed investigation in this aspect will also lead to a correlation between folding limits and structural performance within the framework of the specific manufacturing process and material conditions. The acquired results can also be incorporated into parametric models, exploring the possibilities of producing larger deployable surfaces with multiple folds.

## Figures and Tables

**Table 1:** Comparison between simulation results of different curved-crease models in terms of their individual deformation coefficient factors under exactly same boundary and loading conditions along with each generated curved-crease length.

Type of Curved Crease	Curved-Crease Length (cm)	Deformation Coefficient *ξ*
3-pt NURB curve	12	1.78
Evolved curve	12.24	1.72
3-pt circular arc	12.25	2.11
Catenary	12.1	1.82
Ellipse section	12.76	2.44

## Data Availability

The original contributions presented in the study are included in the article.
